# Consensus Statements on the Approach to COVID-19 Vaccine Allergy Safety in Hong Kong

**DOI:** 10.3389/falgy.2021.690837

**Published:** 2021-07-14

**Authors:** Valerie Chiang, Agnes S. Y. Leung, Elaine Y. L. Au, Marco H. K. Ho, Tak Hong Lee, Adrian Y. Y. Wu, Gary W. K. Wong, Philip H. Li

**Affiliations:** ^1^Division of Clinical Immunology, Department of Pathology, Queen Mary Hospital, Hong Kong, China; ^2^Department of Paediatrics, Prince of Wales Hospital, The Chinese University of Hong Kong, Hong Kong, China; ^3^Department of Paediatrics & Adolescent Medicine, Queen Mary Hospital, The University of Hong Kong, Hong Kong, China; ^4^Allergy Centre, Hong Kong Sanatorium and Hospital, Hong Kong, China; ^5^Centre for Allergy and Asthma Care, Hong Kong, China; ^6^Division of Rheumatology & Clinical Immunology, Department of Medicine, Queen Mary Hospital, The University of Hong Kong, Hong Kong, China

**Keywords:** COVID19, allergy, vaccine, safety, consensus, Hong Kong

## Abstract

**Background:** Mass coronavirus disease 2019 (COVID-19) vaccination to achieve herd immunity is an effective means to mitigate the current COVID-19 pandemic. Reports of COVID-19 vaccine-associated allergic reactions and lack of clear local guidance are contributing factors leading to a low vaccine acceptance rate in the community. A task force of experts from the Hong Kong Institute of Allergy (HKIA) has been formed to address current needs.

**Objective:** To formulate a set of consensus statements (CS) on COVID-19 vaccine allergy safety (VAS) in Hong Kong.

**Methods:** A nominated task force of experts managing patients with drug and vaccine allergies in Hong Kong formulated the CS by the Delphi method. An agreement was *a priori* defined as ≥80% consensus.

**Results:** A total of 11 statements met the criteria for consensus with good overall agreement among task force members, including seven statements on pre-vaccination recommendations and four statements on vaccination and post-vaccination guidance. Individuals with a history of suspected allergic reaction to prior COVID-19 vaccination should not receive further COVID-19 vaccination, and other groups at risk of COVID-19 vaccine-associated allergic reactions have been identified. The importance of pre-vaccination and post-vaccination assessment by frontline healthcare workers and evaluation by allergists are highlighted.

**Conclusion:** The CS provides pragmatic and timely guidance for local frontline healthcare providers on decisions regarding COVID-19 VAS.

## Background

The severe acute respiratory syndrome coronavirus 2 (*SARS-CoV-2*) causing coronavirus disease 2019 (COVID-19) has infected more than 100 million people globally, taken away the lives of more than 2 million individuals, and caused irretrievable damage to society (1). Vaccination is the single most effective way to reduce deaths and severe illness caused by *SARS*-*CoV*-*2*, and mass vaccination of 60–70% of the total population to achieve herd immunity appears to be the most effective public health intervention to control the pandemic (2).

However, even before the commencement of the COVID-19 vaccination program in Hong Kong, the overall vaccine acceptance rate was lower than 40% (3). This low acceptance rate correlated with perceived harm of COVID-19 vaccination and trust (or lack of) in the healthcare system. Likewise, since the start of the COVID-19 vaccination program, there has been much anxiety and fear regarding the possibility of COVID-19 vaccine-associated allergic reactions. This has greatly impacted the confidence of the general public and thus the low rate of vaccine uptake.

Many countries have released their guidelines regarding COVID-19 vaccine allergy safety (VAS), including how to screen people with a higher risk of COVID-19 vaccine-associated allergies and how to approach patients with suspected reactions (4–9). Despite having experienced cases of possible vaccine-associated allergic reactions already, no clear guidance has been made available in Hong Kong regarding COVID-19 VAS so far. Even among healthcare professionals, there have been many uncertainties on how to evaluate and approach this important population-wide dilemma.

In view of this, the Hong Kong Institute of Allergy (HKIA) formed a local task force of experts in the field of Immunology and Allergy to formulate a set of consensus statements (CS) on the pressing issue of COVID-19 VAS in Hong Kong.

## Methods

Consensus statements were formulated by the Delphi method, soliciting the opinions of experts managing patients with drug and vaccine allergies in Hong Kong (10). All members of this task force were nominated representatives of the HKIA and consisted of two academic representatives [PHL and ASYL], two public sector representatives working in the VAS Clinic of the Hospital Authority Hong Kong West Cluster [VC and EYLA], two private sector representatives [TL and AYYW], and two professional body representatives [MHKH and GWKW]. PHL also acted as the facilitator. No ethics approval was required as no human or animal subjects were involved.

In the first Delphi round, a structured questionnaire containing a set of proposed CS encompassing important issues pertaining to COVID-19 VAS in Hong Kong was formulated by the task force. The statements were formed based on various aspects regarding VAS from the pre-vaccination phase (e.g., screening and management of patients at higher risk of COVID-19 vaccine-associated allergies, requirements of medical regulations, and infrastructure), vaccination process (e.g., monitoring requirements, management of potential allergic reactions), and post-vaccination events (e.g., referral of suspected COVID-19 vaccine-associated allergies). In the second round of Delphi, all members of the task force completed the above-described questionnaire *via* an online anonymized system. They were not required to answer all questions and could select “no opinion” to any of the statements. In the third and final round of Delphi, the task force reviewed the aggregated responses of the questionnaires. If further clarification or elaboration on any statements was required, the questionnaire was adapted and sent back to participants with feedback. The term “drugs” was defined to include both non-vaccine and vaccine medications.

Responses were graded as “Strongly Agree,” “Tend to Agree,” “Neither Agree nor Disagree,” “Tend to Disagree,” and “Strongly Disagree” for each respective statement, scoring +1, +0.5, 0, −0.5, and −1, respectively. The consensus was *a priori* defined as 80% or more responses to “Strongly Agree” or “Tend to Agree.” Scores are reported as the mean and SD (scale from +1 to −1). More extreme scores and lower SD, therefore, indicated stronger consensus.

## Results

**Figure 1:**
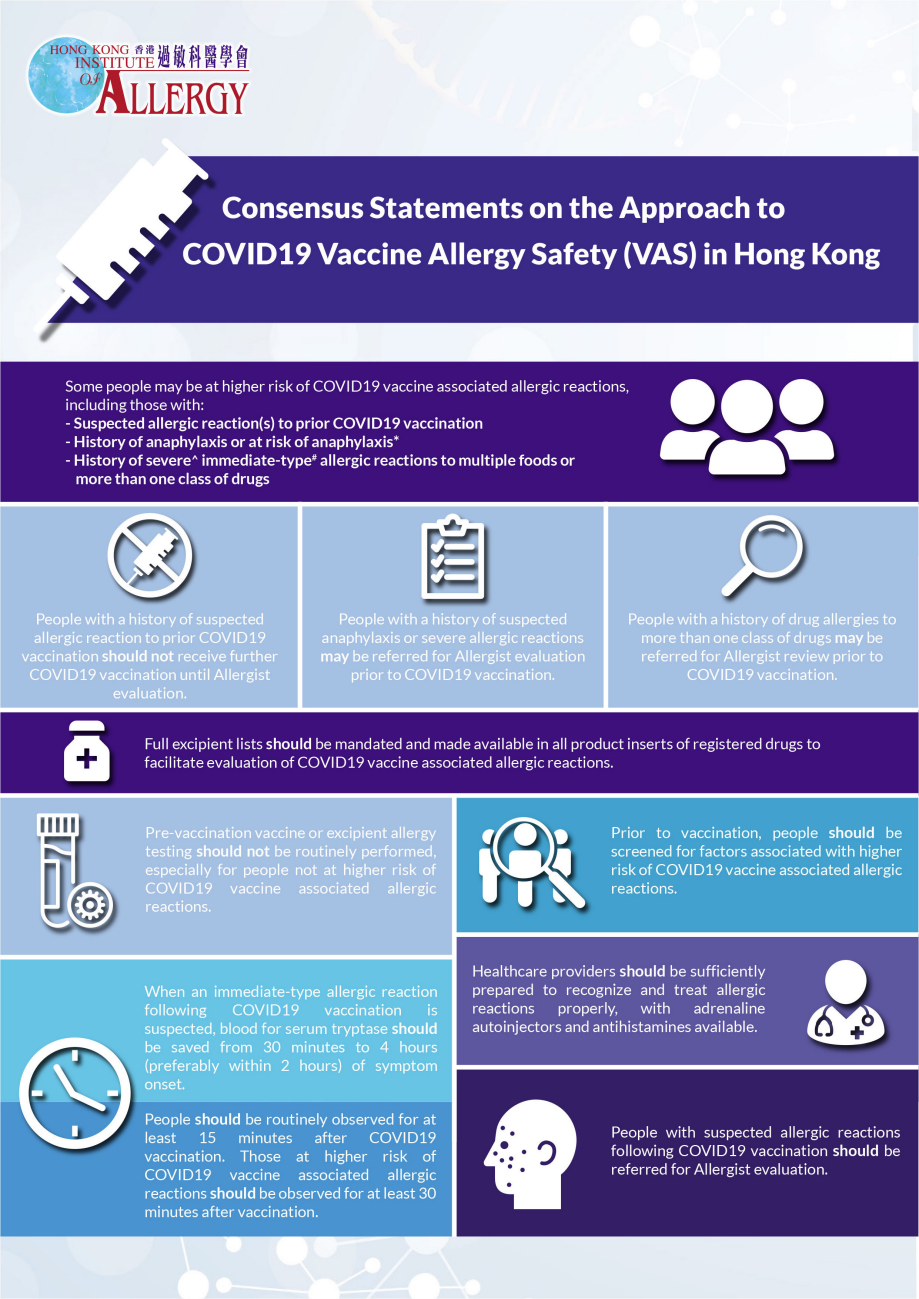
Graphical summary of consensus statements (CS).

A total of 11 statements reached consensus after multiple rounds of Delphi. All members of the task force were invited to further elaborate on the details and suggest refinements to certain statements that reached consensus as deemed necessary. Results after revision following the final round of Delphi were summarized in Table 1, Figure 1, and were as follows:

**Table 1:** Summary of consensus statements (CS).

(1) Some people may be at higher risk of COVID-19 vaccine-associated allergic reactions, including those with: - Suspected allergic reaction(s) to prior COVID-19 vaccination - History of anaphylaxis or at risk of anaphylaxis^*^ - History of severe^∧^ immediate-type^#^ allergic reactions to multiple foods or more than one class of drugs.
(2) People with a history of suspected allergic reaction to prior COVID-19 vaccination should not receive further COVID-19 vaccination until allergist evaluation.
(3) People with a history of suspected anaphylaxis or severe allergic reactions may be referred for allergist evaluation prior to COVID-19 vaccination.
(4) People with a history of drug allergies to more than one class of drugs may be referred for allergist review prior to COVID-19 vaccination.
(5) Full excipient lists should be mandated and made available in all product inserts of registered drugs to facilitate the evaluation of COVID-19 vaccine-associated allergic reactions.
(6) Pre-vaccination vaccine or excipient allergy testing should not be routinely performed, especially for people not at higher risk of COVID-19 vaccine-associated allergic reactions.
(7) Prior to vaccination, people should be screened for factors associated with a higher risk of COVID-19 vaccine-associated allergic reactions.
(8) Healthcare providers should be sufficiently prepared to recognize and treat allergic reactions properly, with adrenaline autoinjectors and antihistamines available.
(9) When an immediate-type allergic reaction following COVID-19 vaccination is suspected, blood for serum tryptase should be saved from 30 min to 4 h (preferably within 2 h) of symptom onset.
(10) People should be routinely observed for at least 15 min after COVID-19 vaccination. Those at higher risk of COVID-19 vaccine-associated allergic reactions should be observed for at least 30 min after vaccination.
(11) People with suspected allergic reactions following COVID-19 vaccination should be referred for allergist evaluation.

* *Anaphylaxis, according to the National Institute of Allergy and Infectious Disease and the Food Allergy and Anaphylaxis Network (NIAID/FAAN) Criteria*.

∧ *Severe, according to modified Ring and Messmer grading, Grade II or above*.

# *Immediate-type, onset of reaction(s) occurred within 1 h following allergen exposure*.

Pre-vaccination:

**CS #1: Some people may be at higher risk of COVID-19 vaccine-associated allergic reactions, including those with:**

- **Suspected allergic reaction(s) to prior COVID-19 vaccination**.
- **History of anaphylaxis or at risk of anaphylaxis**.
- **History of severe immediate-type allergic reactions to multiple foods or more than one class of drugs**.

There was 100% agreement (Score: +0.63 ± 0.23) with CS #1. The definitions of anaphylaxis and severe reactions were based on the National Institute of Allergy and Infectious Disease and the Food Allergy and Anaphylaxis Network (NIAID/FAAN) criteria (Table 2) and modified Ring and Messmer grading (Table 3), respectively (11, 12). Immediate-type reactions were defined as reactions with symptom onset within 1 h following allergen exposure.

**Table 2:** Anaphylaxis according to National Institute of Allergy and Infectious Disease and the Food Allergy and Anaphylaxis Network (11) (NIAID/FAAN).

Anaphylaxis is likely when one of the three criteria is fulfilled:
**1. Acute onset of an illness (minutes to several hours) with involvement of the skin, mucosal tissue**, or both (e.g., generalized hives, pruritus or flushing, and swollen lips-tongue-uvula) **AND at least one of the following:** a. Respiratory compromise (e.g., dyspnoea, wheeze-bronchospasm, stridor, reduced peak expiratory flow, and hypoxemia). b. Reduced blood pressure or associated symptoms of end-organ dysfunction [e.g., hypotonia (collapse), syncope, and incontinence].
**2. Two or more** of the following that occurs rapidly after exposure ***to a likely allergen for that patient*** (minutes to several hours): a. Involvement of the skin mucosal (e.g., generalized hives, itch-flush, and swollen lips-tongue-uvula). b. Respiratory compromise (e.g., dyspnoea, wheeze-bronchospasm, stridor, reduced peak expiratory flow, and hypoxemia). c. Reduced blood pressure or associated symptoms [e.g., hypotonia (collapse), syncope, and incontinence]. d. Persistent gastrointestinal symptoms (e.g., crampy abdominal pain and vomiting).
3. Reduced blood pressure after exposure ***to known allergy for that patient (minutes to several hours)***. - Infants and children: low systolic blood pressure (age-specific) or >30% decrease in systolic blood pressure. - Adults: systolic blood pressure of <90 mmHg or >30% decrease from the baseline of that person.

**Table 3:** Modified ring and messmer grading (12).

**Grade**	**Symptoms**
I	Generalized cutaneous signs: erythema, urticaria, with or without angioedema.
II	Non-life-threatening multivisceral involvement with cutaneous signs, hypotension and tachycardia, and bronchial hyperreactivity.
III	Severe life-threatening multivisceral involvement: collapse, tachycardia or bradycardia, arrhythmias, and bronchospasm.
IV	Cardiac and/or respiratory arrest.

**CS #2: People with a history of suspected allergic reaction to prior COVID-19 vaccination should not receive further COVID-19 vaccination until allergist evaluation**.

There was 100% agreement (Score: +1.00 ± 0.00) with CS #2.

**CS #3: People with a history of suspected anaphylaxis or severe allergic reactions may be referred for allergist evaluation prior to COVID-19 vaccination**.

There was 88% agreement (Score: +0.75 ± 0.53) with CS #3.

**CS #4: People with a history of drug allergies to more than one class of drugs may be referred for allergist review prior to COVID-19 vaccination**.

There was 88% agreement (Score: +0.63 ± 0.52) with CS #4.

**CS #5: Full excipient lists should be mandated and made available in all product inserts of registered drugs to facilitate the evaluation of COVID-19 vaccine-associated allergic reactions**.

There was 88% agreement (Score: +0.81 ± 0.38) with CS #5.

**CS #6: Pre-vaccination vaccine or excipient allergy testing should not be routinely performed, especially for people not at higher risk of COVID-19 vaccine-associated allergic reactions**.

There was 100% agreement (Score: +0.94 ± 0.18) with CS #6.

**CS #7: Prior to vaccination, people should be screened for factors associated with a higher risk of COVID-19 vaccine-associated allergic reactions**.

There was 88% agreement (Score: +0.69 ± 0.70) with CS #7.

Vaccination:

**CS #8: Healthcare providers should be sufficiently prepared to recognize and treat allergic reactions properly, with adrenaline autoinjectors and antihistamines available**.

There was 88% agreement (Score: +0.81 ± 0.37) with CS #8.

**CS #9: When an immediate-type allergic reaction following COVID-19 vaccination is suspected, blood for serum tryptase should be saved from 30 min to 4 h (preferably within 2 h) of symptom onset**.

There was 100% agreement (Score: +0.69 ± 0.37) with CS #9.

**CS #10: People should be routinely observed for at least 15 min after the COVID-19 vaccination. Those at higher risk of COVID-19 vaccine-associated allergic reactions should be observed for at least 30 min after vaccination**.

There was 100% agreement (Score: +1.00 ± 0.00) with CS #10.

Post-vaccination:

**CS #11: People with suspected allergic reactions following COVID-19 vaccination should be referred for allergist evaluation**.

There was 100% agreement (Score: +0.94 ± 0.18) with CS #11.

## Discussion

Patient fear regarding the risk associated with a new vaccine may lead to vaccine hesitancy, the major hurdle in attaining herd immunity. To provide reassurance and support toward the success of the global vaccination effort, we present the first set of CS of Hong Kong on the approach to COVID-19 VAS. Given the novel and emerging nature of COVID-19 vaccination and associated allergic reactions, there is still a paucity of evidence or published literature on such events. Despite this, many authorities in various other countries have also published recommendations mostly based on expert opinion (Table 4). Thus, it is not possible to create an evidence-based treatment guideline, instead, the consensus described in this report comprises the collective expertise of members of the HKIA task force. This is in-line with various recommendations published in other countries (Table 4). It is likely that as more experience becomes available for COVID-19 vaccination, clinical practices will evolve so recommendations will be updated from a background of accumulated evidence. Nonetheless, in the interim, given the pressing and urgent need for such recommendations to facilitate the decisions of healthcare providers about various aspects of COVID-19 VAS in Hong Kong, it was felt essential that a series of CS are produced without delay.

**Table 4:** Summary of recommendations regarding COVID-19 vaccine allergy safety (VAS) in other countries.

**Country**	**Institution**	**Contraindication to vaccination**	**Precaution to vaccination**	**May proceed to vaccination**
Australia (4)	Australasia Society of Clinical Immunology and Allergy	Vaccination contraindicated: •Documented anaphylaxis to one of the ingredients contained in the COVID-19 vaccine to be administered (Pfizer—PEG or Astra Zeneca—Polysorbate 80). •Anaphylaxis to a prior dose of a COVID-19 vaccine. Note: Anaphylaxis with one type of COVID-19 vaccine may not preclude vaccination with another vaccine, but this should only occur if the precautions listed above are met. If there is a high risk of an allergic reaction to one of the vaccines (such as a known allergy to PEG or Polysorbate 80), it may be possible to have another vaccine that does not contain the ingredient, subject to availability and medical advice.	Vaccination with Precautions: •Immediate (within 4 h) and generalized symptoms of a possible allergic reaction without anaphylaxis to a previous dose of a COVID-19 vaccine. •Generalized allergic reaction (without anaphylaxis) to one of the ingredients in the COVID-19 vaccine to be administered (Pfizer-PEG or Astra Zeneca-Polysorbate 80). •Prior history of anaphylaxis to previous vaccines and/or multiple drugs (injectable and/or oral) where ingredients such as PEG or polysorbate 80 may conceivably be the cause. •A known systemic mast cell activation disorder with raised mast cell tryptase, that requires treatment. Precautions: Review or discussion prior to vaccination by a clinical immunology/allergy or vaccinology specialist, to develop a risk/benefit assessment for each patient. •Skin testing to the vaccine and/or graded doses should be considered in some cases. •Vaccination is a medical facility equipped for the management of anaphylaxis (such as a medical clinic with multiple doctors available, or a hospital clinic). •The post-vaccination observation period should be at least 30 min.	Vaccination without additional Precautions: Vaccinate in the community as per national recommendations, with a post-vaccination observation period of 15 min. This includes people with: •History of allergy, including anaphylaxis to food, drugs, venom, or latex. •Allergic conditions, including asthma, atopic dermatitis (eczema), or allergic rhinitis (hay fever).
Canada (5)	Canadian Society of Allergy and Clinical Immunology	Assessment by an allergist is warranted in any individual with a suspected allergy to a COVID-19 vaccine or any of its components. This includes anyone who has experienced a suspected allergic reaction after receiving the first dose of a COVID-19 vaccine, or someone with a suspected or confirmed allergy to a component of the vaccine. A proper assessment will help to clarify whether and how a COVID-19 vaccine can be (re)administered and, if necessary, can help in the selection of an alternative COVID-19 vaccine when one becomes available.		Assessment by an allergist is NOT required for individuals with a history of unrelated allergies, including allergies to foods, drugs, insect venom, or environmental allergens. In these individuals, the available COVID-19 vaccines can be administered without any special precautions. As for the routine administration of all vaccines, they should be administered in a healthcare setting capable of managing anaphylaxis, and individuals should be observed for a minimum of 15–30 min following vaccination.
Singapore (6)	Ministry of Health	The Pfizer-BioNTech COVID-19 Vaccine should not be given to persons with a history of: (1) anaphylaxis or severe allergies—Persons with a history of anaphylaxis which include severe angioedema, bronchospasm and/or hypotension, to other drugs, vaccines, food, insect stings, or unknown trigger (idiopathic) and persons with a history of having been prescribed an Epi-Pen suggests anaphylaxis risk and such persons, (2) severe drug reactions, or (3) an allergic reaction to the first dose of the vaccine or its components.	NSAID-induced angioedema is generally not considered an allergic (type 1 hypersensitivity) reaction. Nevertheless, persons with a history of NSAID-induced angioedema may be at some increased risk of anaphylaxis from the Pfizer-BioNTech COVID-19 vaccine and are advised to defer at this time. However, if the clinical risk/benefit strongly favors vaccination, then the vaccination should be conducted in a healthcare facility with immediate access to anaphylaxis treatment. Persons with multiple allergies without anaphylaxis, who have never had severe angioedema, respiratory or circulatory involvement, maybe at some increased risk of anaphylaxis, and are advised to defer receiving the Pfizer-BioNTech COVID-19 vaccine at this time. However, if the clinical risk/benefit strongly favors vaccination, then the vaccination should be conducted in a healthcare facility with immediate access to anaphylaxis treatment.	Persons with atopy (such as eczema, allergic rhinitis, or well-controlled asthma) can be vaccinated. Although there may be some increased risk of hypersensitivity reactions to the Pfizer-BioNTech COVID-19 Vaccine, these conditions are common and there is not enough evidence to contraindicate the vaccine at this time. A person with a family history (but NOT a personal history) of anaphylaxis CAN be vaccinated.
United Kingdom (7)	Commission on Human Medicines /Medicines and Healthcare products Regulatory Agency	•Hypersensitivity to the active substance or any of the excipients of the vaccine •A second dose of the vaccine should not be given to those who have experienced anaphylaxis the first dose of the vaccine. Precautions for use: Events of anaphylaxis have been reported. Appropriate medical treatment and supervision should always be readily available in case of an anaphylactic reaction following the administration of the vaccine. Close observation for at least 15 min is recommended following vaccination.		Those with any other allergies such as a food allergy can now have the vaccine *(updated on 30 December 2020)*
United States (8)	Centers for Disease Control and Prevention	History of the following: •Severe allergic reaction (e.g., anaphylaxis) after a previous dose or to a component of the vaccine •Immediate allergic reaction of any severity after a previous dose or known (diagnosed) allergy to a component of the vaccine. Actions: •Do not vaccinate. •Consider referral to allergist-immunologist. •Consider other vaccine alternatives.	Among people without a contraindication, a history of: •Any immediate allergic reaction to other vaccines or injectable therapies Actions: •Risk assessment •Consider referral to allergist-immunologist. •30-min observation period if vaccinated.	Among people without a contraindication or precaution, a history of: •Allergy to oral medications (including the oral equivalent of an injectable medication). •History of food, pet, insect, venom, environmental, latex, etc., allergies. •Family history of allergies. Actions: •30-min observation period: people with a history of anaphylaxis (due to any cause). •15-min observation period: all other people
World Allergy Organization (9)	World Allergy Organization	Vaccination contraindicated: •Prior allergic reaction to the vaccine in question. •For an mRNA-based COVID-19 vaccine, prior allergic reaction to another mRNA vaccine. •Prior allergic reaction to a component of the vaccine, including PEG. Actions: •Do not vaccinate with the vaccine in question. •Ideally, choose a different COVID-19 vaccine if available and not contraindicated. •Consider referral to allergist-immunologist.	Special precautions: •History of immediate allergic reactions (usually anaphylaxis) to multiple, different drug classes, with the trigger unidentified (this may indicate PEG allergy). •History of anaphylaxis to a vaccine or a parenteral monoclonal antibody preparation. •History of idiopathic anaphylaxis Mast cell disease. Actions: •Assess risk and possibility of PEG-allergy. •Consider referral to allergist-immunologist. •Consider observation for 30 min if vaccination proceeds. •There are no data to inform recommendations regarding pre-treatment e.g., with an antihistamine. •Pre-treatment with antihistamines may mask initial symptoms of a reaction.	Proceed with vaccination: •Prior history of allergic reaction to an identified food or venom or defined group of medication. •Inhalant allergy. •Family history of allergies. •Local (non-systemic) reaction to prior vaccination. •Hypersensitivity to non-steroidal anti-inflammatory drugs e.g., aspirin, ibuprofen. •Allergen immunotherapy. •Patients with stable asthma on biologics. Actions: •Proceed with vaccination as normal, according to local guidelines •An observation period of 15–30 min may be advisable

CS #1–4 pertains to defining those people at higher risk of potential COVID-19 vaccine-associated allergies. The groups defined in CS #1 are in concordance with the broader guidance published by the Department of Health (DH), which suggests that referral to specialists in Immunology and Allergy for assessment should be considered before vaccination for those with a “history of reactions to multiple classes of drugs or anaphylaxis; history of anaphylaxis or at risk of anaphylaxis; severe reactions to other allergens, e.g., drugs/ foods/ insects (especially injectables) and severe reactions to other vaccines or prior COVID-19 vaccine” (13). In addition to the recommendations of DH, we offer definitions for “anaphylaxis” and “severe reactions” by drawing references to the well-established NIAID/FAAN criteria and Ring and Messmer grading. We also suggest focusing current definitions to describe immediate-type reactions only, given that current recommendations do not pertain to delayed-type vaccine-associated reactions (14).

CS #2–4 provide details on the recommendations on the three different groups described in CS #1. However, it is important to note that except for the history of previous reactions to prior COVID-19 vaccination, the other higher-risk groups were proposed based on pragmatic (rather than purely medical or evidence-based) reasons. Although patients with a history of anaphylaxis have been cautioned against, or even excluded from, COVID-19 vaccination in some countries, we were unaware of any definite evidence that these factors were associated with an increased risk of subsequent COVID-19 vaccine-elicited allergic reactions. Based on current literature, around 31% of patients experiencing anaphylaxis after messenger RNA (mRNA) COVID-19 vaccines had a prior history of anaphylaxis (15). However, it is not known what proportion of patients who have had a prior history of anaphylaxis, experienced an anaphylactic episode following mRNA vaccination. The task force emphasizes the importance of diagnosing “anaphylaxis” based on objective evidence and strict accordance with the NIAID/FAAN criteria. Also, at present, there was insufficient evidence to recommend any specific cut-off regarding the duration for CS #3 (i.e., time since last anaphylaxis event). Future case-control studies will be essential to ascertain if such association truly exists and to guide further refinements to future recommendations.

The inclusion of a “history of severe immediate-type allergic reactions to multiple foods or more than one class of drugs” was designed to encompass patients with potentially undiagnosed (or “idiopathic”) anaphylaxis and excipient allergies. As mentioned, the term “drugs” was defined to include both non-vaccine and vaccine medications. There is limited evidence to suggest that the presence of drug allergy alone is a risk factor for COVID-19 vaccination-associated allergic reactions. Instead, it is thought that allergic reactions to vaccines are primarily due to adjuvants or excipients in the vaccine and not to the active ingredient itself. Excipients have been implicated as the major allergen for many COVID-19 vaccine-associated allergic reactions, especially following mRNA vaccines (16). It would, therefore, be prudent to avoid vaccinating those with known allergies to the same excipients. However, this is extremely difficult given the current medical infrastructure. In Hong Kong, drugs are not required legally to include excipient lists in product inserts. Therefore, it is near impossible to diagnose a potential excipient allergy if the culprit excipient cannot be identified in the causative drug or vaccine (17). Furthermore, given the unmet provision of allergy services and specialists in Hong Kong, anaphylaxis survivors remain severely underinvestigated and underdiagnosed (18, 19). For example, among Hong Kong adult patients, more than 10% of anaphylaxis survivors did not have an identifiable cause for their allergies and were mistakenly labeled with a diagnosis of multiple food allergies (20).

To prevent patients with excipient allergies to be inadvertently reexposed to the same excipients in COVID-19 vaccines, we advise patients with a history of severe immediate-type allergic reactions to multiple foods or multiple classes of drugs to seek the evaluation of allergists prior to vaccination. Therefore, CS #5 was instigated in hope that full excipient lists in all registered drugs could become mandated in the future so that more people could be safely vaccinated after a proper and comprehensive evaluation.

Skin testing has been a well-established tool for diagnosing various excipient allergies and suggested mRNA COVID-19 vaccine allergy testing (16, 21). However, excipient allergy skin testing, especially with polyethylene glycol (PEG), is not without risk and has been associated with systemic allergic reactions (22). Furthermore, the predictive values of either vaccine- or excipient-based skin testing remain uncertain and carry a risk of false-positive or negative results. Given the potential risks and uncertainties of excipient allergy testing, there was 100% agreement among the task force for CS #6 which explicitly warns that pre-vaccination vaccine or excipient allergy testing should not be routinely performed. Instead, as stated in CS #7, we recommend that those planning to receive COVID-19 vaccination should be screened for factors associated with a higher risk of COVID-19 vaccine-associated allergic reactions (as described previously) based on their clinical history. Specialized allergy testing should be reserved for those deemed at higher risk of COVID-19 vaccine-associated allergic reactions after allergist evaluation and appropriate counseling.

CS #8–10 pertains to VAS throughout the vaccination process, including monitoring requirements and management of potential allergic reactions. Again, these CS are generally in line with the broader DH guidance and current practice for anaphylaxis care. CS #8 supports the Hong Kong Anaphylaxis Consortium recommendation that “adrenaline autoinjectors should be used as first-line treatment for all patients at risk of anaphylaxis” (23). Although autoinjectors could be replaced with adrenaline ampoules (suggested by one task force member who voted “Neither Agree/Disagree” to CS #8), the task force agreed that autoinjectors may limit the risk of incorrect dosing and preferred over ampoules in the community setting if resources allow. An acute serum tryptase level can be of immense benefit to support a diagnosis of anaphylaxis in the subsequent allergy consultation. Therefore, CS #9 reminds all frontline healthcare providers to save the blood for serum tryptase from 30 min to 4 h (preferably within 2 h) of symptom onset when a severe immediate-type allergic reaction following COVID-19 vaccination is suspected. CS #10 recommends routine post-vaccine observation for at least 15 min and extended to 30 min for those at higher risk of COVID-19 vaccine-associated allergic reactions, which arbitrarily balances between safety and practicality. Reassuringly, a 30-min observation period would cover 90% of previously reported anaphylaxis cases based on recently published experiences (15).

Last, but not least, CS #11 emphasizes the importance of an allergist referral for all people with suspected allergic reactions following COVID-19 vaccination. Patients with suspected COVID-19 vaccine-associated allergies must withhold further vaccination prior to appropriate specialist evaluation. If a certain vaccine (or vaccine component) allergy is suspected or confirmed, the allergist may advise further testing and/or propose the use of a suitable alternative vaccine. Optimal allergy testing strategies are still undergoing investigation and are beyond the scope of this document. Despite the persistently low allergist to population ratio of Hong Kong, this has improved slightly in recent years with the advent of new allergist trainees and allied health services (18). Allergy centres are now available in both the public and private sectors, and both local medical universities have dedicated formal allergy-focused teaching and research. A publicly available list of accredited Immunologists and allergists can also be found in the Specialist Register of the Medical Council of Hong Kong (24).

Coronavirus disease 2019 vaccination and approach to VAS is a novel and emerging field of medicine accompanied by challenges and opportunities. With the accumulation of global data and expertise, these statements will likely be superseded by more robust and specific evidence-based guidance soon. Meanwhile, we hope the present CS can help facilitate healthcare providers to make important decisions regarding COVID-19 VAS in Hong Kong.

## Data Availability Statement

The original contributions presented in the study are included in the article/supplementary material, further inquiries can be directed to the corresponding author/s.

## Author Contributions

VC, MH, GW, and PL contributed to conception and design of the study. PL organized the Delphi Process. VC and PL performed the statistical analysis and wrote the first draft of the manuscript. VC, AL, and PL wrote sections of the manuscript. All authors contributed to manuscript revision, read, and approved the submitted version.

## Conflict of Interest

AW is a consultant of ALK-Abelló A/S and director of Ksena Healthcare Ltd. The remaining authors declare that the research was conducted in the absence of any commercial or financial relationships that could be construed as a potential conflict of interest.
